# Nanomagnetite-embedded PLGA Spheres for Multipurpose Medical Applications

**DOI:** 10.3390/ma12162521

**Published:** 2019-08-08

**Authors:** Valentina Grumezescu, Oana Gherasim, Irina Negut, Stefan Banita, Alina Maria Holban, Paula Florian, Madalina Icriverzi, Gabriel Socol

**Affiliations:** 1 Lasers Department, National Institute for Lasers, Plasma, and Radiation Physics, 077125 Magurele, Romania; 2 Department of Science and Engineering of Oxide Materials and Nanomaterials, Faculty of Applied Chemistry and Materials Science, Politehnica University of Bucharest, 011061 Bucharest, Romania; 3 Microbiology & Immunology Department, Faculty of Biology, University of Bucharest, 77206 Bucharest, Romania; 4 Ligand-Receptor Interactions Department, Institute of Biochemistry, Romanian Academy, 060031 Bucharest, Romania

**Keywords:** magnetic nanoparticles, biopolymeric spheres, hyperthermia, antimicrobial materials, multifunctional materials

## Abstract

We report on the synthesis and evaluation of biopolymeric spheres of poly(lactide-co-glycolide) containing different amounts of magnetite nanoparticles and Ibuprofen (PLGA-Fe_3_O_4_-IBUP), but also chitosan (PLGA-CS-Fe_3_O_4_-IBUP), to be considered as drug delivery systems. Besides morphological, structural, and compositional characterizations, the PLGA-Fe_3_O_4_-IBUP composite microspheres were subjected to drug release studies, performed both under biomimetically-simulated dynamic conditions and under external radiofrequency magnetic fields. The experimental data resulted by performing the drug release studies evidenced that PLGA-Fe_3_O_4_-IBUP microspheres with the lowest contents of Fe_3_O_4_ nanoparticles are optimal candidates for triggered drug release under external stimulation related to hyperthermia effect. The as-selected microspheres and their chitosan-containing counterparts were biologically assessed on macrophage cultures, being evaluated as biocompatible and bioactive materials that are able to promote cellular adhesion and proliferation. The composite biopolymeric spheres resulted in inhibited microbial growth and biofilm formation, as assessed against *Staphylococcus aureus*, *Pseudomonas aeruginosa,* and *Candida albicans* microbial strains. Significantly improved antimicrobial effects were reported in the case of chitosan-containing biomaterials, regardless of the microorganisms’ type. The nanostructured composite biopolymeric spheres evidenced proper characteristics as prolonged and controlled drug release platforms for multipurpose biomedical applications.

## 1. Introduction

The personalized therapy desideratum aims for an accurate and controllable local distribution of therapeutic agents, and to ensure the specific and selective targeting of the molecular or cellular receptors [1,2]. Besides some general requirements (such as biocompatibility, biodegradability, and non-immunogenicity) [3,4,5], unconventional pharmaceutical formulations must also fulfil several specific requirements, including bioavailability and bioactivity, accurate pharmacological profiles, maximal therapeutic effects, and minimal or absent side effects [6,7,8].

Nanoparticle-based drug formulations have proved their potential to deal with challenging diseases. A simple search in the main research publication data bases revealed an impressive number of studies performed on nanoparticles designed for drug delivery applications in 2018 (5563 in Pubmed [9], 29,789 in Scopus [10], and 9165 in ScienceDirect [11]; entries searched in July 2019). Nanoparticles have revolutionized how drugs are formulated, delivered, and explored for specific and selective therapies, and they are currently utilized in diagnosis, cancer therapy, HIV and AIDS therapy, nutraceutical delivery, anti-bacterial systems, etc. [12,13,14].

Polymer particles or capsules are proper choices for modern pharmacotherapy, thanks to their intrinsic physicochemical versatility and tunable functionality, which enable higher loading and encapsulation efficiency of various therapeutics (such as phytochemical and drug molecules, proteins, growth factors, genes, and cells) and controllable or/and triggerable release mechanisms [15,16].

In particular, micro-/nanoparticles based on poly(lactic-co-glycolic) acid (PLGA) own a leading position in the design and implementation of nanotechnology-derived pharmacological formulations. By adjusting the molecular weight of lactide and glycolide constituents and their ratio, as well as the size, morphology, and microstructure of resulted copolymer, intrinsic features of PLGA-based biomaterials (e.g., solubility, biodegradability, metabolization, drug release profile, delivery efficiency) could be tuned for performant pharmaceutics [17,18]. Among PLGA copolymers, the 50:50 representative has been extensively investigated for bioactive formulations, owing to its beneficial features such as water-uptake rate, degradation kinetics, and morphological modifications [19,20].

The successful experimental use of unconventional pharmaceutical formulations based on this particular PLGA copolymer was reported in modern strategies for diabetes-related conditions [21,22], HIV management [23,24], vaccine therapy [25], therapeutic angiogenesis [26,27], and tissue engineering [28,29,30]. Relevant examples of PLGA-based anti-infective strategies include treatment of streptococcal infections [31], ocular infection management [32], treatment of vaginal infection [33], and systemic antibiotherapy [34]. Also, remarkable results were reported in designing novel and superior platforms for cancer treatment, including anti-angiogenic therapy [35], selective and targeted chemotherapy [36,37], and externally triggered chemotherapy [38,39,40].

Among nanomaterials, magnetite particles (Fe_3_O_4_) have gained remarkable attention for novel therapeutic alternatives, thanks to their facile and large yield synthesis and peculiar nanosize-related characteristics, including versatile physicochemical properties, functionalization potential, and tunable biocompatibility [41,42]. Moreover, Fe_3_O_4_ with core dimensions of 10–20 nm have superparamagnetic behavior. Magnetically-responsive biomaterials embedded with Fe_3_O_4_ nanoparticles have been assessed as multifunctional systems for specific, selective, and performance-enhanced anti-infective [43,44] and anti-cancer applications [45,46], as well as for regenerative medicine [47,48]. In particular, Fe_3_O_4_-based nanostructured systems are promising platforms for successful controlled and targeted drug delivery applications by means of magnetic hyperthermia [41,49,50].

The incorporation of magnetic nanoparticles into biopolymeric microspheres represents an attractive strategy for modern pharmacotherapy, since the additional functionality relies on guiding nanostructured spheres with an external magnetic field gradient and triggering the release of bioactive compounds [51,52]. The applications of magnetic nanoparticle–embedded microspheres include detection and diagnosis by magnetic resonance imaging, and local pharmacological effect by subjecting the nanostructured microspheres to magnetic hyperthermia [53,54,55]. Following the exposure of such nanostructured platforms to external high amplitude alternating magnetic field, targeted and controlled drug delivery may be achieved. This synergistic therapeutic effect is attained by the local thermally-induced pathophysiological alteration of organic macromolecules (which occurs due to important relaxations of both magnetic moments and particles within particles’ crystalline structure and surrounding medium, respectively) and the enhanced drug release kinetics (which occurs due to the heat-induced release of bioactive molecules and denaturation or degradation of biopolymeric matrix) [56,57,58].

Chitosan (CS) is an attractive candidate for modern pharmaceutical formulations, thanks to its peculiar ability to improve the stability and solubility of bioactive molecules and to its intrinsic mucoadhesive, antioxidant, analgesic, haemostatic, non-immunogenic, antimicrobial [59], and antitumor effects [60,61,62]. The proposed mechanisms for intrinsic anti-pathogenic effects exhibited by CS-based biomaterials include electrostatic interactions between cationic CS and negatively-charged surface of pathogenic cells [63,64], interactions with microbial nucleic acids [65,66], and chelation of microbial structures and essential nutrients containing metallic ions [67,68]. Moreover, along with its great intrinsic antimicrobial properties, CS is known as an active agent to improve the efficiency of antibiotics [69,70], and recent pharmaceutical formulations rely on the use of this biopolymer in the fight of resistant microorganisms [71].

Ibuprofen (iso-butyl-phenyl-propionic acid) (IBUP) is a non-steroidal anti-inflammatory drug (NSAID), introduced as an alternative to conventional Aspirin treatment. The pharmacological activity of IBUP (analgesic, anti-inflammatory, and antipyretic effects) is related to the inhibition of cyclooxygenase COX2 enzyme, which is further related to the synthesis of prostaglandins (vasodilating lipid compounds that inhibit platelet aggregation) and thromboxane A (vasoconstrictive lipid molecule that stimulate platelet aggregation) [72,73]. Since the expression of COX2 was reported during tumorigenesis, the specific inhibition of this enzyme can be explored for selective destruction of cancer cells [74,75]. Also, the antimicrobial activity of IBUP was demonstrated both in vitro and in vivo (in a mouse animal model), and the clinical administration of this anti-inflammatory drug seemed to improve the outcome of severe respiratory infections, such as cystic fibrosis. Moreover, this drug has been proven to be highly efficient against relevant opportunistic Gram-negative bacteria recognized as multidrug resistant species, such as *P. aeruginosa* and *Burkholderia sp.* [76].

In this study, we developed multifunctional composite microspheres for drug delivery applications. Given the selected drug molecule and the addition of nanomagnetite, PLGA-Fe_3_O_4_-IBUP nanostructured spheres represent potential candidates for local drug delivery applications, being useful tools for anti-inflammatory or anti-cancer approaches. Moreover, PLGA-CS-Fe_3_O_4_-IBUP spheres (obtained by the addition of CS) extend the potential use of our nanostructured biopolymeric carriers towards modern anti-infective therapy.

## 2. Materials and Methods

### 2.1. Materials

All chemicals required for the synthesis of nanostructured composites were purchased from Sigma-Aldrich Chemie GmbH (Taufkirchen, Germany), namely anhydrous ferric chloride (FeCl_3_, >99.99% trace metal basis), ferrous sulphate heptahydrate (FeSO_4_·7H_2_O, >98%), Ibuprofen (United States Pharmacopeia testing specifications, small molecule), chitosan (non-animal derived, low molecular weight, high purity) and poly(D,L-lactide-co-glycolide) copolymer (PLGA) with 50:50 lactide-to-glycolide molar ratio. Ammonium hydroxide solution (25% NH_3_ in H_2_O), chloroform (CHCl_3_), analytical graded acetone (C_6_H_6_O), and ethanol (C_2_H_6_O), as well as all reagents required for the obtaining of simulated body fluid (SBF), such as NaCl, NaHCO_3_, KCl, K_2_HPO_4_·3H_2_O, MgCl_2_·6H_2_O, HCl, CaCl_2_, Na_2_SO_4_, and (CH_2_OH)_3_CNH_2_, were also purchased from Sigma-Aldrich.

Sigma-Aldrich also provided most reagents used during biological and microbiological assays, including phorbol 12-myristate 13-acetate (PMA), paraformaldehyde (PFA), bovine serum albumin (BSA), and Triton X-100. RPMI 1640 cell culture medium, fetal bovine serum (FBS), and streptomycin/penicillin antibiotic mixture were purchased from Gibco (Thermo Fisher Scientific, Waltham, MA, USA). MTS assay was acquired from Promega Company (Madison, WI, USA), while red-labeled Alexa Flour—488 Phalloidin and blue-fluorescent DAPI stain were purchased from Invitrogen (Thermo Fisher Scientific, Carlsbad, CA, USA).

### 2.2. Synthesis Methods

#### 2.2.1. Synthesis of Fe_3_O_4_ Nanoparticles

For the synthesis of Fe_3_O_4_ nanoparticles, we applied the wet chemical co-precipitation method. According to our optimized and previously described protocol [77,78], spherical-shaped and nanosized Fe_3_O_4_ particles could be thus obtained.

#### 2.2.2. Synthesis of Fe_3_O_4_-Embedded Biopolymeric Spheres

The synthesis of biopolymeric spheres embedded with Fe_3_O_4_ nanoparticles and loaded with IBUP, PLGA-Fe_3_O_4_-IBUP, was made by the microemulsion technique [79,80]. Mixtures consisting of PLGA:IBUP (with 10:1 mass ratio) and different amounts of magnetite nanopowder (10, 20, and 50 mg) were dispersed in CHCl_3_, followed by the addition of a 2% (w/v) polyvinyl alcohol (PVA) solution. The as-obtained composite mixtures were subjected to the sonication process for 6 min, which was performed in ON/OFF steps of 6/3 s at a temperature of 37 °C by using a SONIC-1200WT sonicator (MRC Scientific Instruments, Harlow, Essex, UK). The resulted emulsions were added to deionized water and continuously stirred until complete solvent evaporation. The final emulsions were subjected to a triple centrifuge washing treatment and collected synthesis products were subsequently lyophilized.

Depending on the amount of Fe_3_O_4_ added during the synthesis, PLGA-Fe_3_O_4_-IBUP biopolymeric spheres were denoted IBUP10, IBUP20, and IBUP50, corresponding to PLGA:Fe_3_O_4_:IBUP mass ratios of 10:0.5:1, 10:1:1, and 10:2.5:1, respectively.

The same microemulsion protocol was used to obtain drug-free nanostructured microspheres (PLGA-Fe_3_O_4_, denoted as S) and drug-loaded nanostructured composite microspheres of PLGA and CS (PLGA-CS-Fe_3_O_4_-IBUP systems were obtained by adding 1 wt% of CS solution), which were considered during biological and microbiological assays.

### 2.3. Physicochemical Investigation

#### 2.3.1. Transmission Electron Microscopy (TEM)

The TEM data were collected by using the Tecnai^TM^ G2 F30 S-TWIN high resolution transmission electron microscope from FEI Company (Hillsboro, OR, USA). The specific point and line resolutions of the microscope are 2 and 1 Å, respectively. Before TEM investigation, a small amount of the Fe_3_O_4_ sample was dispersed in ethanol, sonicated for 15 min, placed onto the carbon-coated cooper grid, and dried at room temperature.

#### 2.3.2. Scanning Electron Microscopy (SEM)

SEM analysis revealed morphological features of nanostructured biopolymeric spheres, both after synthesis and after dynamic drug release studies. The samples were investigated by an Inspect S scanning electron microscope (FEI Company, Eindhoven, The Netherlands) at an acceleration voltage of 20 kV. Prior to SEM analysis, all samples were capped with a thin gold layer in order to diminish the accumulation of electric charges on their surface.

#### 2.3.3. Fourier-Transform Infrared Spectroscopy (FT-IR)

FT-IR analysis was performed to investigate the stoichiometry and chemical function integrity of the biopolymeric spheres, both after synthesis and after dynamic drug release studies. In this respect, a FTIR 8400s spectrophotometer (Shimadzu Europa GmbH, Duisburg, Germany) was used. The spectral collection was recorded in transmission mode in the range of 5000–500 cm^−1^, at 4 cm^−1^ resolution, with 40 individual scans being acquired for each sample.

#### 2.3.4. Ultraviolet-Visible Spectrophotometry (UV-Vis)

In order to evaluate the release profiles of IBUP, both after dynamic and external activated drug release studies, UV-Vis analysis was performed by an Evolution 220 spectrophotometer (Thermo Fisher Scientific, Darmstadt, Germany). The absorption spectra of supernatants resulted after drug release studies were recorded in the range of 200–400 nm. For qualitative analysis, we monitored the absorption bands at 265 and 274 nm, which are characteristic for IBUP molecules in the UV-Vis region [81,82].

### 2.4. Drug Release Study

#### 2.4.1. Drug Release under Dynamic Conditions

In order to evaluate the behavior of PLGA-Fe_3_O_4_-IBUP systems under dynamic biological simulated conditions, a multichannel bioreactor connected to a peristaltic pump was used. For these drug release experiments, simulated body fluid (SBF, pH = 7.4) was prepared after Kokubo’s recipe and was selected as the active testing medium thanks to the ion concentrations, which are similar to the human blood plasma [83,84].

By using the same amount of lyophilized powders, suspensions of PLGA-Fe_3_O_4_-IBUP spheres in SBF were obtained. A volume of 4 mL from PLGA-Fe_3_O_4_-IBUP suspensions was introduced into each bioreactor channel, followed by their testing in dynamic mode, with 1 mL/min flow rate. After dynamic testing periods (7, 14, and 21 days), solutions from each channel were collected. A small volume of each resulted solution was dropped onto silicon substrates for subsequent FT-IR analysis and SEM investigation. The remaining collected solutions were centrifuged for 20 min at 6000 rpm (MRC Scientific Instruments) and the as-obtained supernatants were analyzed by UV-Vis.

#### 2.4.2. Drug Release under External Activation

**Figure 1:**
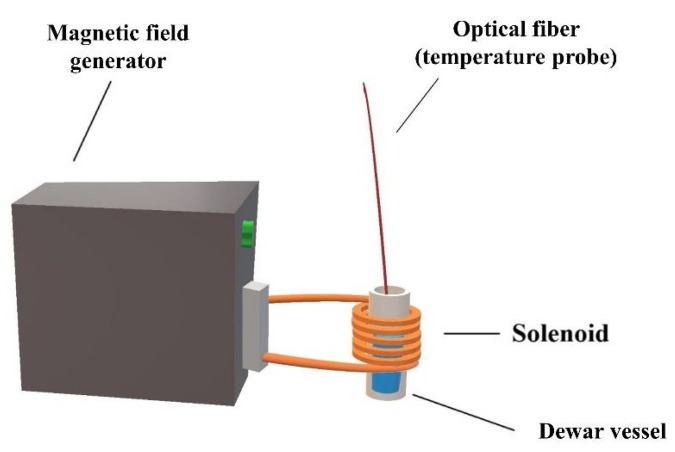
Schematic representation of the experimental setup used during hyperthermia tests.

The potential use of PLGA-Fe_3_O_4_-IBUP nanostructured spheres in hyperthermia applications was assessed by using a heating system model Ultra Heat S Series (RF) with 2 kW maximum output power from UltraFlex Power Technologies (Sofia, Bulgaria) (Figure 1). The customized experimental setup used during our experiments corresponds to literature descriptions [85], and implies the sample subjection to a radiofrequency magnetic field and monitoring the thermal response. The liquid sample was introduced into a Dewar vial, which was further placed inside the water-cooled copper solenoid (a 925 nH inductance coil).

The PLGA-Fe_3_O_4_-IBUP solutions, obtained by dispersing ~14 mg of lyophilized microspheres in deionized water, were introduced into the Dewar vial and subjected to three magnetic fields, with powers representing 60%, 80%, and 100% from the maximum output power. The temperature evolution of each sample was recorded up to 50 °C (slightly above the 42–45 °C temperature range associated with the occurrence of apoptosis and necrosis of cancer cells) [49,86,87] by using an optical fiber supplied with a TS4 sensor (precision ± 0.2 °C) from Optocon (Dresden, Germany) connected to a PC. In all experiments, deionized water was used as blank specimen.

The information regarding the notation and composition of PLGA-Fe_3_O_4_-IBUP systems and their corresponding testing parameters under external magnetic fields are included in Table 1.

### 2.5. Biological Evaluation

Following the performed drug release studies, we identified the most promising PLGA-Fe_3_O_4_-IBUP systems for biological assays. For comparison, we also considered drug-free PLGA-Fe_3_O_4_ spheres (further denoted as S) and drug-loaded composite microspheres of PLGA and CS (PLGA-CS-Fe_3_O_4_-IBUP). For biological assays, nanostructured biopolymeric spheres were transferred as coatings onto titanium substrates (12 mm diameter and 0.1 mm thickness) by using the immersion (dip-coating) method [88,89]. Before using, all samples were sterilized by immersion in 1% streptomycin/penicillin solution for 15 min.

#### 2.5.1. Cell Cultures

Human THP-1 cells (ATCC^®^ TIB-202™) were maintained in RPMI 1640 medium with 10% (v/v) inactivated fetal bovine serum (FBS) and 1% (v/v) streptomycin/penicillin at 37 °C in a humidified atmosphere of 5% CO_2_. For in vitro biological assessment, THP-1 cells were cultured onto material surfaces at a density of 4 × 10^5^ cells/surface material in 24-well plates (Nunc). Macrophages were generated from monocytic THP-1 cells by incubation for 72 h with 100 ng/mL of phorbol 12-myristate 13-acetate (PMA).

#### 2.5.2. Cell Viability

The proliferation of THP-1 cells cultured on the material surface was evaluated by the MTS assay (CellTiter 96^®^ AQueous One Solution Cell Proliferation Assay), which is based on the reduction of a tetrazolium compound, (3-(4,5-dimethylthiazol-2-yl)-5-(3-carboxymethoxyphenyl)-2-(4-sulfophenyl)-2H-tetrazolium), to insoluble formazan crystals by a dehydrogenase present in the metabolically active cells. The amount of formazan released into the culture medium is proportional to the number of live cells. Macrophage-differentiated THP-1 cells were incubated with MTS solution at 37 °C. After 15 min, 100 μL of supernatant was transferred to a 96-well plate and the optical density was measured at 450 nm using a Mithras Berthold LB940 microplate reader (Berthold Technologies, Bad Wildbad, Germany).

#### 2.5.3. Cell Adhesion and Morphology

The effect of composite nanostructured materials on macrophage morphology and adhesion was investigated by fluorescence microscopy, following the distribution of actin filaments. Macrophages attached to surfaces (3 days after differentiation) were fixed for 15 min with 4% PFA, permeabilized with 0.2% Triton X-100, blocked for 1 h with 0.5% BSA-PBS mixture, and then washed with PBS. Actin filaments were stained with red-labeled Alexa Fluor 488 Phalloidin for 1 h at room temperature in 0.5% BSA-PBS solution. The nuclei were counterstained with blue-fluorescent DAPI (4′,6′-diamidino-2-phenylindole dihydrochloride) for 1 min at room temperature. After repeated washing with PBS, samples were mounted on microscope slides with ProLong Gold antifade (Molecular Probes, Eugene, OR, USA; Life Technologies, Carlsbad, CA, USA), an agent that allows the fluorescence signal to be maintained over a prolonged period. The as-treated samples were examined using the 20× and 40× lenses of the Zeiss Axiocam ERc5s Apotom microscope with ApoTome.2 cursor mode and AxioVision4.8 software (Zeiss, Oberkochen, Germany).

### 2.6. Microbiological Evaluation

In order to evaluate the antimicrobial effects exhibited by nanostructured biopolymeric spheres, we only considered the most promising PLGA-Fe_3_O_4_-IBUP systems and their CS-containing corresponding counterparts, namely PLGA-CS-Fe_3_O_4_-IBUP. Similar to the biological protocol, coatings of nanostructured biopolymeric spheres were obtained onto titanium substrates. Prior to microbiological assays, samples were sterilized following 30 min of UV exposure.

#### 2.6.1. Microbial Strains and Growth Conditions

*Staphylococcus aureus* ATCC 25923, *Pseudomonas aeruginosa* ATCC 27853, and *Candida albicans* ATCC 10231 strains were purchased from American Type Culture Collection (ATCC, Manassas, VA, USA). Glycerol stocks were streaked on LB agar (bacteria) and Sabouraud agar (*C. albicans*) to obtain 24 h cultures to be used for all further studies.

#### 2.6.2. Development of the Planktonic Cultures

Sterile dip-coated and reference Ti substrates were added in sterile 6-well plates in 2 mL of LB broth (for bacteria) or Yeast Peptone Glucose (YPG) broth (for yeast) and inoculated with ~10^6^ CFU (colony forming units)/mL of microbial suspensions. The samples were allowed to grow at 37 °C for 24 h. After this step, 200 µL of resulting cultures were transferred in 96-well plates in order to read the absorbance of the obtained cultures using a bench spectrophotometer.

#### 2.6.3. Biofilm Development

Monospecific biofilm development was assessed at different exposure times, using sterile 6-well plates (Nunc). Sterile modified and bare Ti samples were added in plates with 2 mL of LB of YPG broths (for bacteria and yeast, respectively) and inoculated with ~10^6^ CFU/mL of microbial suspensions. The samples were allowed to incubate at 37 °C for three time points (24 h, 48 h, and 72 h) to assess the time dynamic of developed biofilms. After incubation, Ti discs were carefully washed with sterile saline buffer to remove any unattached microbial cells and then immersed in 1 mL sterile saline buffer in Eppendorf tubes to precede biofilm detachment by vigorous vortexing. The resulting biofilm-detached cell suspensions were further diluted and 10 µL of each serial dilution were plated in triplicate on LB agar.

After 24 h of incubation at 37 °C, viable count was performed and the CFU/mL values for each sample were obtained.

## 3. Results and Discussion

### 3.1. PLGA-Fe_3_O_4_-IBUP Biopolymeric Spheres

**Figure 2:**
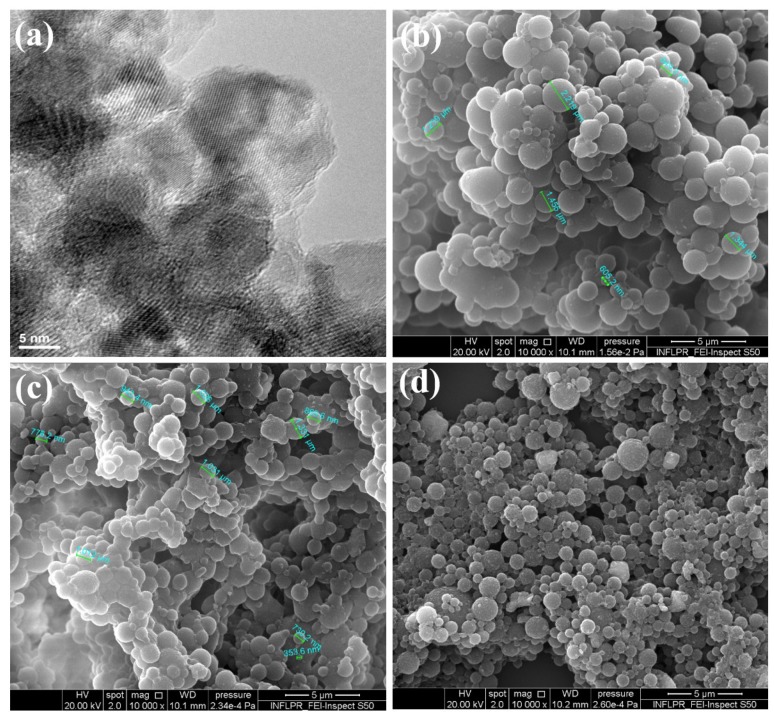
TEM image of (**a**) Fe_3_O_4_ particles, and SEM images of (**b**) IBUP10, (**c**) IBUP20, and (**d**) IBUP50 biopolymeric spheres.

As it can be noticed in the TEM micrograph (Figure 2a), the modified co-precipitation synthesis method enabled the formation of nanosized magnetite particles (mean particle size below 10 nm), with preferential spheroidal shape. 

The microstructure of the PLGA-Fe_3_O_4_-IBUP composite systems obtained by microemulsion protocol by the addition of 10, 20, and 50 mg of magnetite nanopowder (corresponding to IBUP10, IBUP20, and IBUP50 samples, respectively) was investigated by SEM. The collected micrographs are included in Figure 2.

According to SEM micrographs, the microemulsion method resulted in the synthesis of well-defined and individual composite systems with exclusive spherical morphology; the presence of unembedded magnetite nanoparticles is not evidenced at this level. All PLGA-Fe_3_O_4_-IBUP spheres possess sub-micron or micron sizes with comparable dimensions (ranging between ~600 nm and ~2 μm), regardless the amount of Fe_3_O_4_ used during the synthesis process. However, relevant microstructural aspects could be noticed depending on to the presence of different amounts of Fe_3_O_4_. In the case of IBUP10 and IBUP20 samples (Figure 2a,b, respectively), one can notice a prevalent smooth surface and a narrower dimensional distribution of microspheres. One can assume that by using smaller amounts of nanomagnetite, a reduced nanosize-related aggregation tendency could result in complete and uniform embedding of magnetic nanoparticles within polymeric matrices. By contrast, IBUP50 microspheres (Figure 2c) possess a rather textured surface and have more heterogenic dimensions. In this case, which corresponds to the use of a larger amount of Fe_3_O_4_ powder, a predominant agglomeration tendency could determine the increased amounts of nanoparticle aggregates, which could not have sufficient polymer matrix for uniform embedding.

### 3.2. Drug Release Study under Dynamic Conditions

In order to evidence the physicochemical modifications occurred within PLGA-Fe_3_O_4_-IBUP spheres assessed under biological simulated dynamic conditions for 7, 14, and 21 days, drop casted samples were investigated.

**Figure 3:**
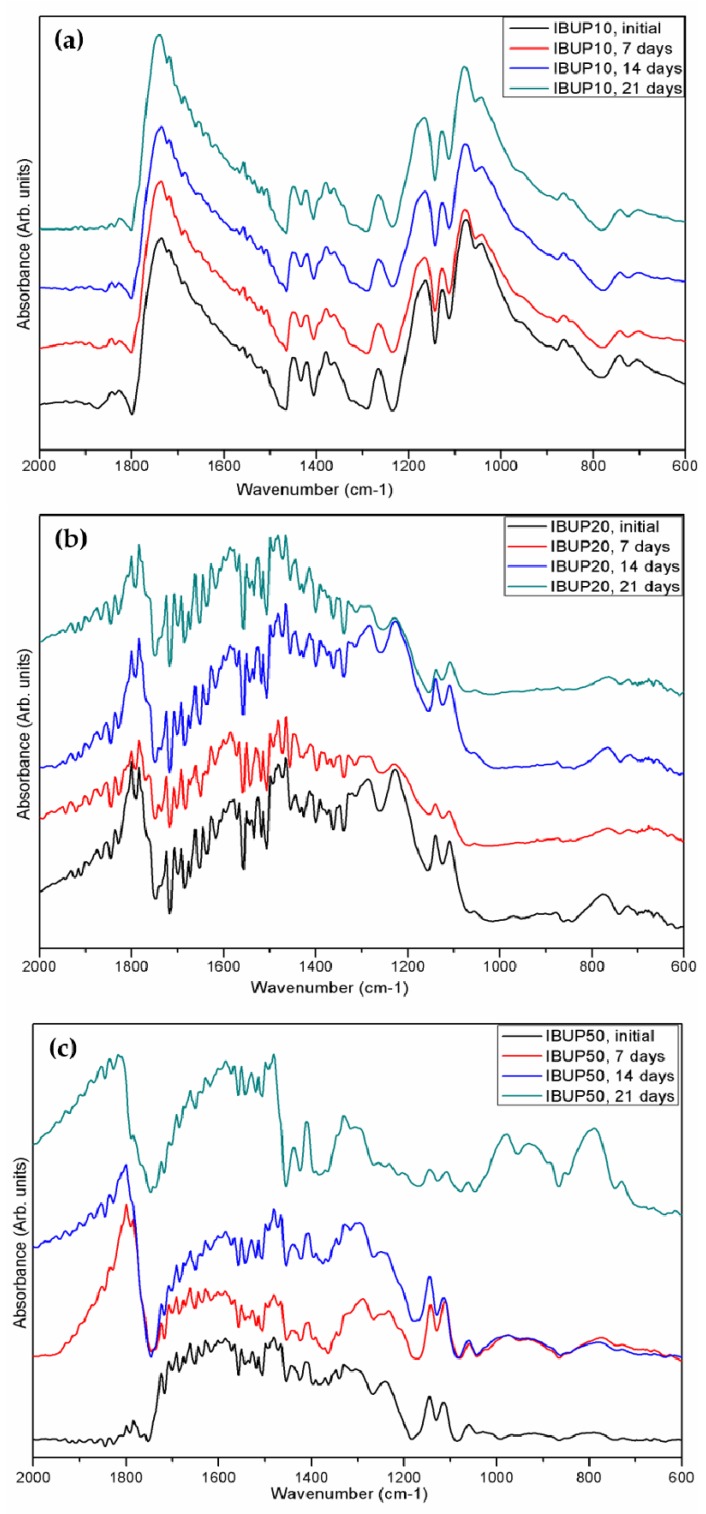
IR spectra of (**a**) IBUP10, (**b**) IBUP20, and (**c**) IBUP50 biopolymeric spheres before and after dynamic tests.

For what concerns the stoichiometry and compositional evolution of the PLGA-Fe_3_O_4_-IBUP nanostructured microspheres, the IR spectra corresponding to samples collected after dynamic tests were compared to IR spectra of initial samples, the results being presented in Figure 3.

As mentioned above, the PLGA-Fe_3_O_4_-IBUP drop cast specimens were obtained by using reduced volumes from collected solutions. Therefore, the recorded IR data evidence noisy spectra, as a direct consequence of the reduced thickness of composite films. However, relevant qualitative information regarding the behavior of PLGA-IBUP-Fe_3_O_4_ spheres during dynamic tests can be obtained from FT-IR results (Figure 3).

In the case of IBUP10 systems (Figure 3a), one can observe the presence of a strong absorption maxima at the wavenumber value of ~1750 cm^−1^, which is resulted from the overlapping stretching vibrations of the carbonyl group present in the aldehyde and ketone groups of PLGA [90,91] and in the carboxylic acid of IBUP [92,93]. At a closer look, a bidentate aspect of those maxima is noticed, which confirms the dual source of C=O vibrations. The double-humped maxima identified at ~1450 cm^−1^ corresponds to asymmetric –CH_3_ and symmetric –CH_2_ stretching within lactic and glycolic units, respectively. Also, at the wavenumber value of ~1390 cm^−1^, one can identify the absorption maxima attributed to both weak stretching vibrations of –OH (from IBUP’s carboxylic groups) and medium-strong wagging vibrations of –CH_2_ groups (corresponding to PLGA copolymer). Furthermore, IR bands identified at ~1320 cm^−1^ mark the presence of C–O asymmetric stretching and C–H bending vibrations within the copolymer, while IR maxima from ~1130 cm^−1^ can be attributed to the symmetrical and asymmetrical stretching vibrations of C–O–C functional groups of glycolic and lactic units [94,95]. In the case of IBUP20 and IBUP50 spheres (Figure 3b,c, respectively), the IR maxima corresponding to the carbonyl functions are shifted towards higher wavenumbers. Moreover, all previously identified IR bands are present in IBUP20 and IBUP50 samples, but with corresponding blueshifted values. Such effects can be related to the decrease of bond lengths, which might have resulted from the increased contribution of electrostatically-bonded nanomagnetite.

The IR spectra of initial microspheres indicate the successful embedding of Fe_3_O_4_ nanoparticles and IBUP molecules within the copolymer matrix, and confirm the synthesis of PLGA-Fe_3_O_4_-IBUP nanostructured biopolymeric spheres. The main IR maxima were preserved within dynamically evaluated microspheres. Since the IR spectra evidence only quantitative changes (namely, increased or decreased absorbance intensity), we can state that PLGA-Fe_3_O_4_-IBUP microspheres possess moderate hydrolytic degradation following their dynamic evaluation in SBF.

**Figure 4:**
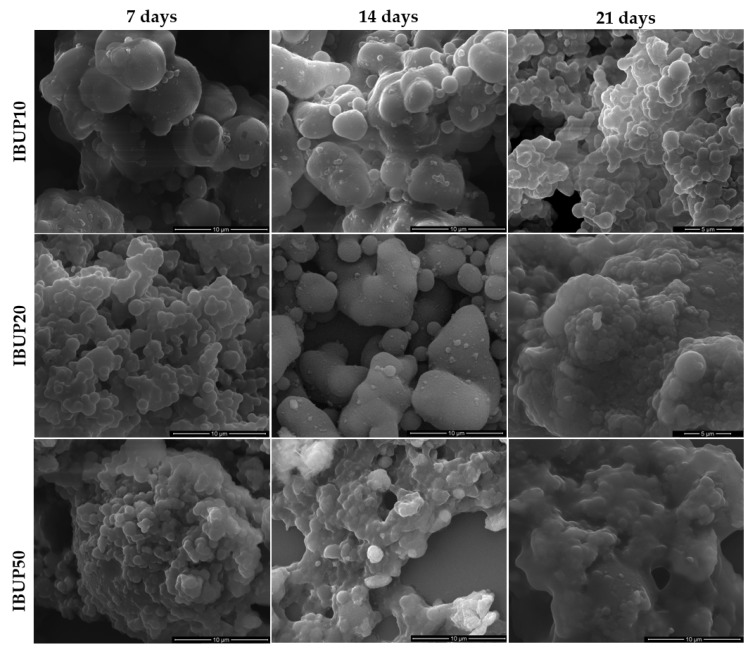
SEM images of IBUP10, IBUP20, and IBUP50 biopolymeric spheres after dynamic tests.

For complementary data regarding the effects of dynamic evaluation on PLGA-Fe_3_O_4_-IBUP spheres, drop casted samples were structurally investigated and the corresponding SEM micrographs are included in Figure 4.

As it can be observed from Figure 4, the evaluation of PLGA-Fe_3_O_4_-IBUP microspheres under biologically simulated dynamic conditions led to the presence of interparticle connections, regardless the amount of nanomagnetite used during synthesis. In all samples, the morphological deformation of composite microspheres and the formation of irregular shaped structures are noticed, the effects being more prominent with the increase of the testing time. Interestingly, in the case of IBUP20 and IBUP50, morphological modifications are more pronounced. Such phenomena may be related to the increased amount of Fe_3_O_4_ nanoparticles, which might have contributed, with hydroxyl-guided interactions [96], to the hydrolysis of PLGA matrix. In the case of IBUP10, SEM images reveal a progressive increase of interparticle junctions with the increasing testing time, but morphological changes occurred after 21 days of dynamic evaluation are less qualitative. In comparison to IBUP20 and IBUP50, IBUP10 biopolymeric spheres maintain a sphere-like shape after the considered prolonged testing interval. Such behavior may be related to the reduced amount of nanomagnetite, as well as to the less significant hydrolytic degradation of PLGA matrix (as evidenced in FT-IR studies).

At this point, it should be noted that IBUP10 (PLGA-Fe_3_O_4_-IBUP microspheres with the lowest content of Fe_3_O_4_ nanoparticles) do not exhibit dramatic morphological modifications after their prolonged evaluation under dynamic conditions.

**Figure 5:**
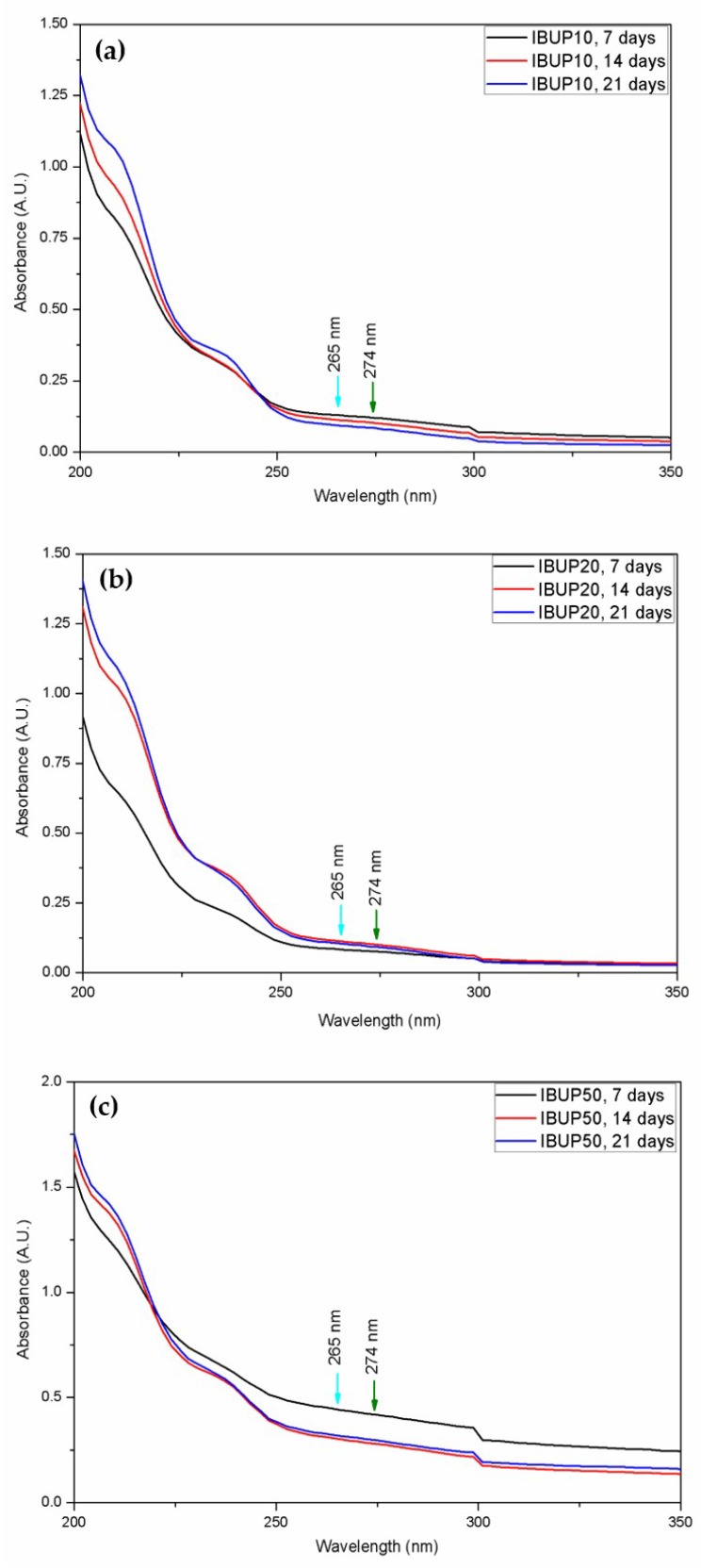
UV-Vis spectra of (**a**) IBUP10, (**b**) IBUP20, and (**c**) IBUP50 biopolymeric spheres after dynamic tests.

The UV-Vis spectrophotometric results corresponding to PLGA-Fe_3_O_4_-IBUP supernatants resulted after dynamic testing are shown in Figure 5.

The qualitative interpretation of UV-Vis results was made by considering the specific IBUP absorbance maxima at 265 and 274 nm (cyan and green arrow, respectively). As shown, the presence of IBUP absorption maxima can be slightly noticed as a wide humped region, regardless the amount of embedded Fe_3_O_4_ nanoparticles. By these results, one can assume that an insignificant release of IBUP from composite microsystems occurred for up to three weeks.

The evaluation of PLGA-Fe_3_O_4_-IBUP microspheres assessed under biomimetic and dynamic conditions evidenced no dramatic alterations for up to three weeks, with a particular emphasis on IBUP10 biopolymeric nanostructured systems (which exhibited minimal compositional changes and preserved sphere-like morphology). Moreover, the UV-Vis qualitative analysis suggests a prolonged release profile of the therapeutic agent. At this point, one can state that the nanostructured biopolymeric microspheres can be used for the incorporation and prolonged release of active substances under dynamic conditions that simulate the biological environment.

### 3.3. Drug Release Study under External Activation

We further assessed the potential use of PLGA-Fe_3_O_4_-IBUP microspheres in hyperthermia applications and aimed to identify the suitable composition that provides maximal effect for future safe investigations. During hyperthermia experiments, the thermal response of our samples could not be monitored higher than 46 °C (regardless of the microspheres’ composition), due to the completely redistributed spatial organization of nanomagnetite-embedded samples inside the Dewar vessel.

**Figure 6:**
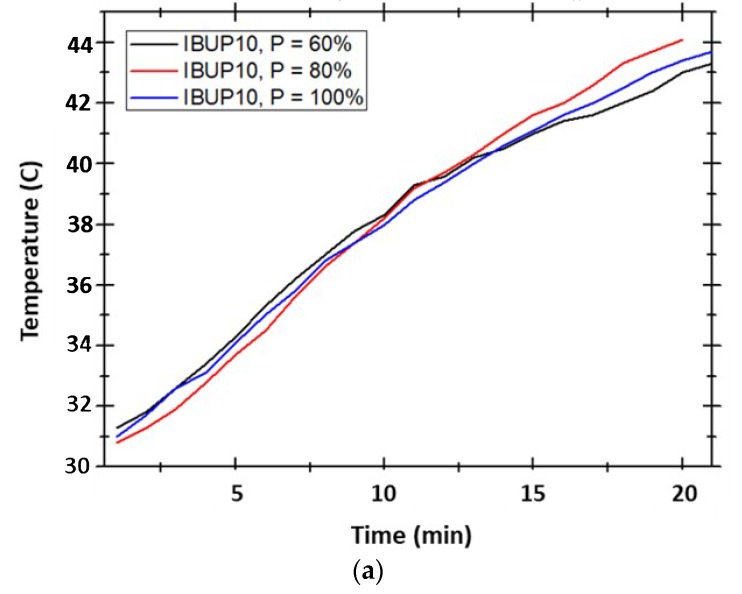

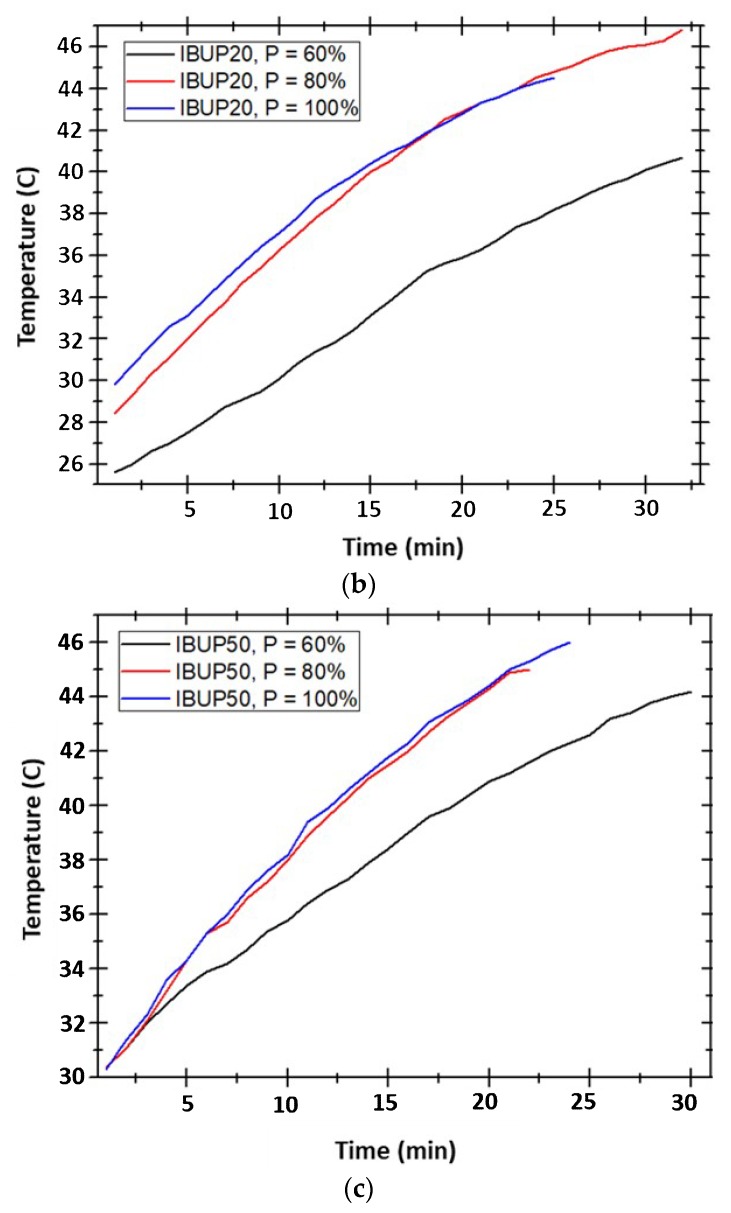
Temperature evolution of PLGA-Fe_3_O_4_-IBUP biopolymeric spheres in the presence of different magnetic fields. (**a**) IBUP10; (**b**) IBUP20; (**c**) IBUP50.

The temperature evolution of PLGA-Fe_3_O_4_-IBUP over time, in the presence of magnetic fields with applied powers of 1.2, 1.6, and 2 kW (corresponding to P = 60, 80 and 100% from maximum output power, respectively), is presented in Figure 6. Within IBUP20 and IBUP50 nanostructured systems, maximum temperatures reached by applying the lowest power (P = 60%) were ~40 °C and 44 °C, respectively; these values were recorded after 30 min of high amplitude alternating field exposure. For these nanostructured biopolymeric spheres, similar thermal responses are attained by applying magnetic fields with 1.6 and 2 kW powers. After 25 min of exposure to radiofrequency magnetic field, the temperature reaches a maximum of ~45 °C, regardless the content of nanomagnetite. A particular case is represented by IBUP10 nanostructured microspheres, in which the heating effect is comparable, regardless the power of external magnetic field. In this situation, a maximum temperature of ~43 °C is attained only after 20 min of exposure.

In order to obtain relevant data regarding the release of IBUP from composite microspheres after high amplitude magnetic field exposure, the UV-Vis absorption spectra of PLGA-Fe_3_O_4_-IBUP-resulted supernatants were collected and compared with the data corresponding to initial nanostructured systems.

**Figure 7:**
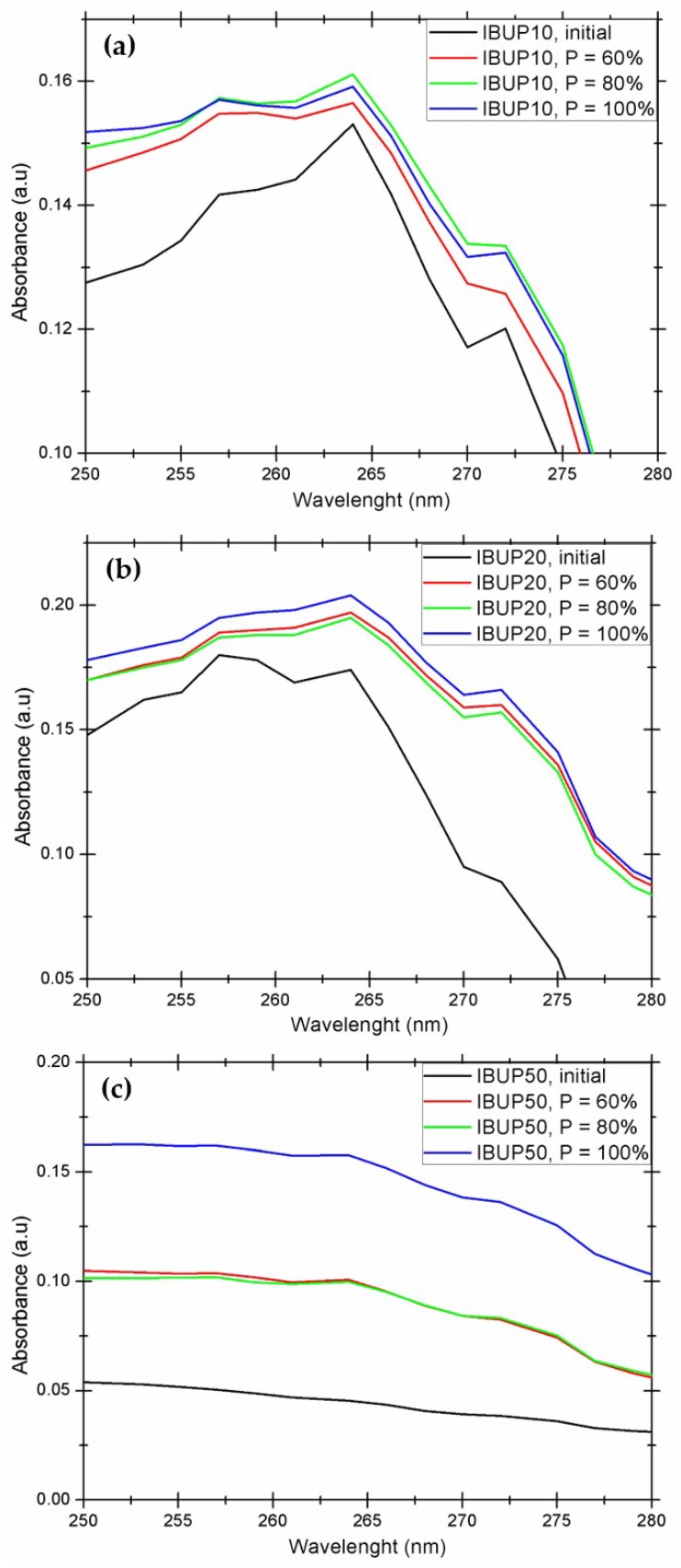
UV-Vis spectra of PLGA-Fe_3_O_4_-IBUP systems resulted after hyperthermia tests. (**a**) IBUP10; (**b**) IBUP20; (**c**) IBUP50.

As it can be noticed from Figure 7, all PLGA-Fe_3_O_4_-IBUP-resulted supernatants possess the specific absorption maxima of IBUP (265 and 274 nm), regardless of the amount of nanomagnetite and the strength of the applied magnetic field. The most relevant responses, in terms of IBUP release after electromagnetic field exposure, are evidenced in the case in IBUP10 and IBUP20 samples. By comparing these nanostructured systems, one can notice that the most prominent drug release effect was assigned to the IBUP20 system exposed to 100% magnetic field strength, followed by the IBUP10 system exposed to the radiofrequency magnetic field with lowest strength (60% of maximum output power).

By considering results reported after subjecting the PLGA-Fe_3_O_4_-IBUP nanostructured systems to external magnetic fields, we noticed that IBUP10 microspheres exhibited a relevant hyperthermia effect, in terms of heating efficiency and drug release under moderate external stimulation. At this point, one can state that IBUP10 biopolymeric spheres are suitable for further analysis.

By gathering the experimental data resulted from drug release studies, we concluded that IBUP10 biopolymeric spheres are potential candidates for both prolonged release of active substances under biomimetically simulated conditions and triggered release of biosubstances under external activation. Therefore, we decided to consider only IBUP10 nanostructured microspheres for biological and microbiological assessment.

### 3.4. Biological Evaluation of Nanostructured Biopolymeric Spheres

#### 3.4.1. Cellular Viability

The viability of THP-1 monocytes grown on materials with thin films of nanomagnetite-embedded biopolymeric spheres of drug-free PLGA (S), IBUP-loaded PLGA (IBUP10), or IBUP-loaded PLGA-CS (IBUP10 CS) was evaluated by MTS colorimetric assay 24 h and 72 h after the differentiation process to macrophages occurred (48 h stimulation with PMA). THP-1 cells differentiated to macrophages were used because monocytes/macrophages have a key role in the inflammatory process [97].

**Figure 8:**
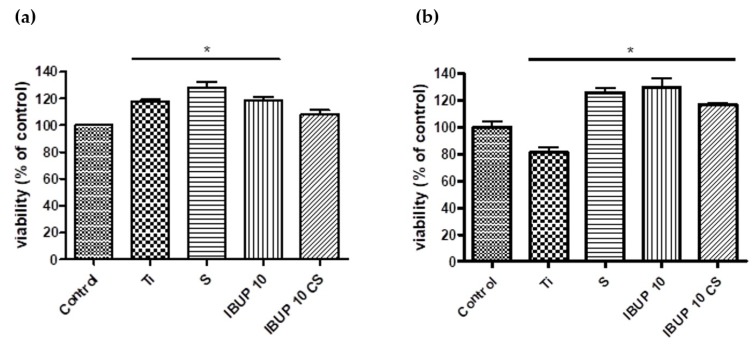
Viability of THP-1 cells attached to the surface of composite biopolymeric spheres coatings after (**a**) 24 h and (**b**) 72 h (data are presented as mean values ± SD and significance was determined at * *p* < 0.05).

As shown in Figure 8, there is no cytotoxic effect of either of thin films of nanostructured biopolymeric spheres tested on differentiated THP-1 cells grown for 24 h or 72 h, as compared to coverslip (control). The results reveal good cellular viability when differentiated macrophages were cultivated in the presence of IBUP10 or IBUP10 CS samples, suggesting an enhanced adherence.

#### 3.4.2. Cellular Adhesion and Morphology

The cell interactions that occur at the interface of an implant are important in providing a closer look at the biocompatibility of materials used in healthcare practice.

The direct effect of nanostructured biopolymeric coatings on the adhesion and morphology of differentiated THP-1 cells was evaluated by fluorescence microscopy. It was of interest to investigate direct material-differentiated macrophage interactions and to observe whether subtle modification of composite’s surface can induce morphological modification of differentiated macrophages that could lead to the initiation of the inflammatory process.

Briefly, actin, a key protein involved in cell adhesion, was investigated and the distribution of actin filaments was compared in the samples with cells grown on thin films of drug-free (S) and drug-loaded (IBUP10 and IBUP10 CS) biopolymeric spheres versus cells grown on coverslip control.

**Figure 9:**
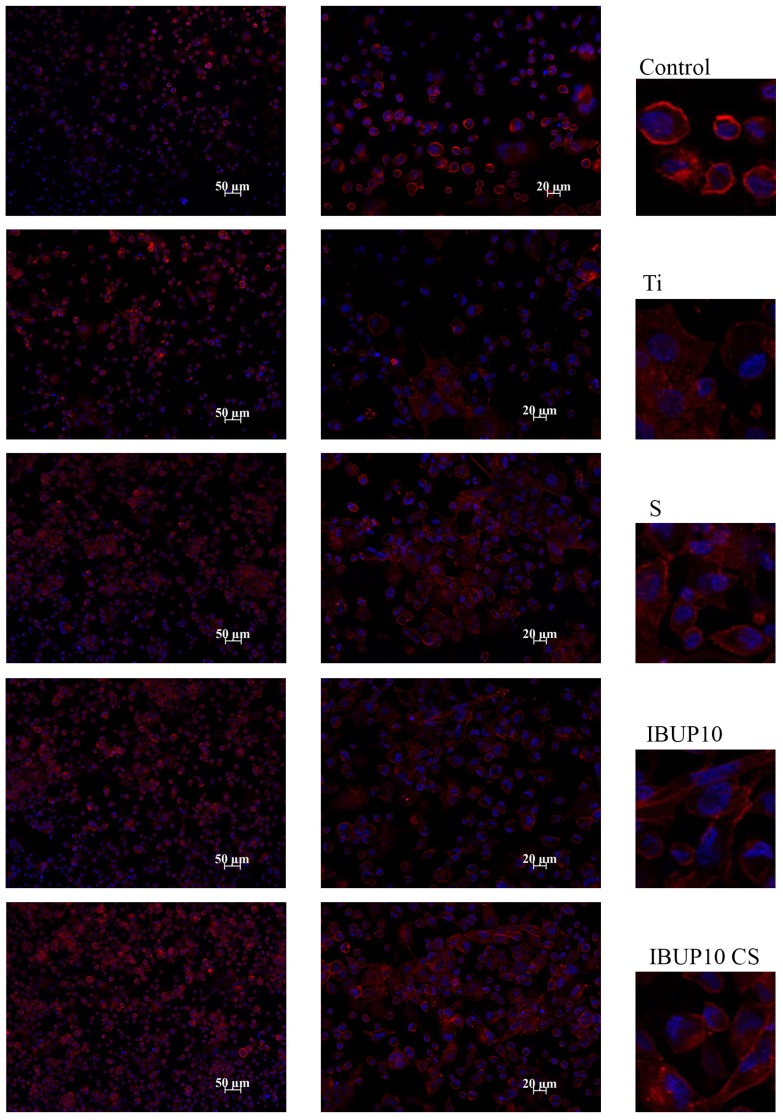
Adhesion and distribution of differentiated THP-1 cells on coatings of composite biopolymeric spheres. Representative images of the attached cells obtained by fluorescence microscopy (40×) by marking actin filaments (red) and nuclei (blue).

Representative fluorescence micrographs are presented in Figure 9. Cells exhibit typical round shape (similar to undifferentiated monocytes morphology) when cultivated on either coverslips or bare Ti substrates. When cells were cultivated on S-coated, IBUP10-coated, or IBUP10 CS-coated Ti samples, a significant increase in cell adhesion was observed. A slight increase in the cell elongation was observed, the effect being more prominent in the case of coatings of IBUP10 and IBUP10 CS biopolymeric spheres. This could be interpreted as an adaptation of cells to surface characteristics. However, morphological modifications induced by composite materials are not associated with an inflammatory response (as evidenced by the absence of TNF-α release by cells grown on these materials; data not shown).

Furthermore, we can suggest that cells have a preferential affinity for substrates modified with biopolymeric spheres, as this conclusion is supported by the polymerized actin filaments (red) present in all samples cultivated on nanostructured biopolymeric composites.

### 3.5. Microbiological Evaluation of Nanostructured Biopolymeric Spheres

For the antimicrobial evaluation of the obtained coatings we considered opportunistic bacterial strain models (Gram-positive *S. aureus* and Gram-negative *P. aeruginosa*) and one yeast model (*C. albicans*).

Although IBUP is known as an anti-inflammatory pharmaceutical agent, recent studies have investigated its efficiency as an antimicrobial agent [98].

**Figure 10:**
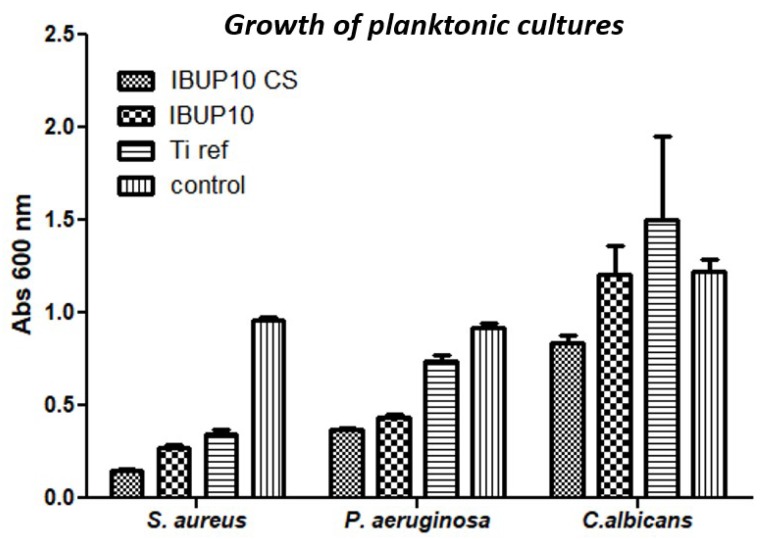
Growth evaluation of planktonic cultures of *S. aureus*, *P. aeruginosa,* and *C. albicans* in the presence of coatings of composite biopolymeric spheres.

The results of microbial planktonic growth obtained in the presence of Ti modified with nanostructured biopolymeric microspheres are included in Figure 10. As it can be observed, the planktonic cultures of tested microbial strains are significantly inhibited in the presence of coatings containing either IBUP or CS and IBUP. Significant inhibition of planktonic growth is observed in the case of IBUP10-coated specimens, when compared to uncoated Ti samples. This effect could be associated to the release of IBUP in the liquid media and to the subsequent antimicrobial activity. The mere addition of CS within the biopolymeric spheres results in the most prominent inhibitory effects against planktonic growth, independent of the microbial strain. It is worth mentioning that the lowest inhibitory effects are observed against *C. albicans*, regardless the type of nanostructured materials. However, the planktonic growth of both bacterial strains is significantly inhibited in the presence of IBUP10 and IBUP10 CS biomaterials, with a slightly increased efficiency being reported in the case of Gram-positive pathogen.

**Figure 11:**
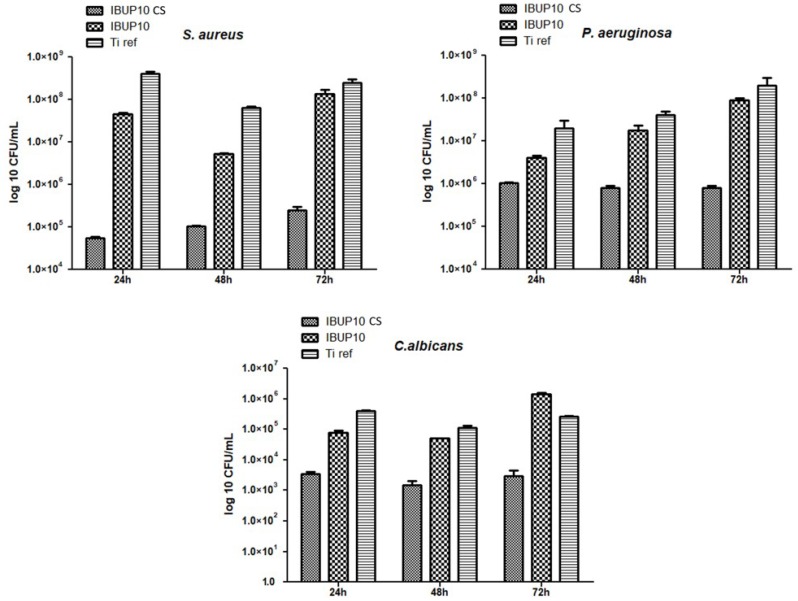
Dynamic biofilm formation ability of tested microbial species in the presence of composite biopolymeric spheres coatings.

Regarding biofilm formation assay, results demonstrate that the biofilm development is especially inhibited by CS-containing coatings (Figure 11), regardless the microorganism’s type. When compared to bare substrates, Ti coated with IBUP10 CS spheres exhibit significant and sustained effects against the formation and development of microbial biofilm. In a parallel way with previously discussed results, the most prominent inhibitory effects are exhibited against the contamination and colonization stages of *S. aureus*. However, coatings containing plain IBUP- encapsulated PLGA nanostructured microspheres also exhibit slight biofilm inhibition ability. This effect is especially manifested in the case of *P. aeruginosa*, supporting recent findings which demonstrate the antimicrobial activity of IBUP against Gram-negative strains [76].

## 4. Conclusions

Biopolymeric microspheres embedding magnetite nanoparticles and Ibuprofen (a common therapeutic drug with acknowledged anti-inflammatory property and recently demonstrated antimicrobial activity) were assessed as platforms for multiple biomedical applications.

A prevalent smooth surface and a narrower dimensional distribution were reported in the case of IBUP10 and IBUP20 microspheres (synthesized by using reduced amounts of nanomagnetite), whereas a rather textured surface and more heterogenic dimensions were noticed for the IBUP50 spheres. The infrared studies confirmed the successful embedding of Fe_3_O_4_ and IBUP molecules within the copolymer matrix of composite microspheres.

The long-term (21 days) biomimetic dynamic evaluation of nanostructured microspheres revealed preserved compositional and structural integrity of composite materials, and suggested prolonged release of IBUP. The exposure of PLGA-Fe_3_O_4_-IBUP composites to different external radiofrequency magnetic fields evidenced important hyperthermia effects, accompanied by significant drug release. A particular case was reported for composite nanosystems embedded with the lowest amount of nanomagnetite (IBUP10), which resulted in the maximal thermal response and the most relevant drug release, after only 20 min of exposure to the weakest alternating filed.

The IBUP10 nanostructured microspheres and their chitosan-containing counterparts (IBUP10 CS) were used as coatings, and proved important promoting effects on the adhesion and proliferation of macrophages.

Both planktonic and dynamic biofilm development of *Staphylococcus aureus*, *Pseudomonas aeruginosa,* and *Candida albicans* strains were inhibited by the composite biopolymeric sphere coatings. A particular prominent antimicrobial effect was exhibited by CS-containing biomaterials against the Gram-positive pathogen.

The composite biopolymeric spheres proposed in our study possess multifunctional features, since their subsequent evaluation may include platforms for controlled and triggered therapy of severe diseases, but also coatings for implantable devices intended for chronic condition management.

## Figures and Tables

**Table 1:** Sample abbreviations and parameters used in hyperthermia experiments.

Sample Code	PLGA:Fe_3_O_4_:IBUP (Mass Ratio)	Applied Power (P% of 2 kW)	Effective Power (W)	Current Intensity (A)	Current Voltage (V)	Frequency (kHz)
IBUP10	10:0.5:1	60	708	9.2	76.95	325.5
IBUP20	10:1:1	80	1088	9.2	109.9	325.5
IBUP50	10:2.5:1	100	1360	9.2	137.37	322

## References

[1] Davoodi P., Lee L.Y., Xu Q., Sunil V., Sun Y., Soh S., Wang C.H. (2018). Drug delivery systems for programmed and on-demand release. Adv. Drug Deliv. Rev..

[2] Hossen S., Hossain M.K., Basher M.K., Mia M.N.H., Rahman M.T., Uddin M.J. (2019). Smart nanocarrier-based drug delivery systems for cancer therapy and toxicity studies: A review. J. Adv. Res..

[3] Habibi N., Kamaly N., Memic A., Shafiee H. (2016). Self-assembled peptide-based nanostructures: Smart nanomaterials toward targeted drug delivery. Nano Today.

[4] Tandon B., Magaz A., Balint R., Blaker J.J., Cartmell S.H. (2018). Electroactive biomaterials: Vehicles for controlled delivery of therapeutic agents for drug delivery and tissue regeneration. Adv. Drug Deliv. Rev..

[5] Barclay T.G., Day C.M., Petrovsky N., Garg S. (2019). Review of polysaccharide particle-based functional drug delivery. Carbohydr. Polym..

[6] Zhu Y., Liao L. (2015). Applications of Nanoparticles for Anticancer Drug Delivery: A Review. J. Nanosci Nanotechnol..

[7] Cagel M., Tesan F.C., Bernabeu E., Salgueiro M.J., Zubillaga M.B., Moretton M.A., Chiappetta D.A. (2017). Polymeric mixed micelles as nanomedicines: Achievements and perspectives. Eur. J. Pharm. Biopharm..

[8] Jacob J., Haponiuk J.T., Thomas S., Gopi S. (2018). Biopolymer based nanomaterials in drug delivery systems: A review. Mater. Today Chem..

[9] PubMed. www.ncbi.nlm.nih.gov/pubmed.

[10] Scopus. www.scopus.com.

[11] Science Direct. www.sciencedirect.com.

[12] Rizvi S.A.A., Saleh A.M. (2018). Applications of nanoparticle systems in drug delivery technology. Saudi Pharm. J..

[13] Patra J.K., Das G., Fraceto L.F., Ramos Campos E.V., del Pilar Rodriguez-Torres M., Acosta-Torres L.S., Diaz-Torres L.A., Grillo R., Swamy M.K., Sharma S. (2018). Nano based drug delivery systems: Recent developments and future prospects. J. Nanobiotechnol..

[14] Lombardo D., Kiselev M.A., Caccamo M.T. (2019). Smart Nanoparticles for Drug Delivery Application: Development of Versatile Nanocarrier Platforms in Biotechnology and Nanomedicine. J. Nanomater..

[15] Shishir M.R.I., Xie L., Sun C., Zheng X., Chen W. (2018). Advances in micro and nano-encapsulation of bioactive compounds using biopolymer and lipid-based transporters. Trends Food Sci. Technol..

[16] George A., Shah P.A., Shrivastav P.S. (2019). Natural biodegradable polymers based nano-formulations for drug delivery: A review. Int. J. Pharm..

[17] Swider E., Koshkina O., Tel J., Cruz L.J., de Vries I.J.M., Srinivas M. (2018). Customizing poly(lactic-co-glycolic acid) particles for biomedical applications. Acta Biomater..

[18] Ding D., Zhu Q. (2018). Recent advances of PLGA micro/nanoparticles for the delivery of biomacromolecular therapeutics. Mater. Sci. Eng. C Mater. Biol. Appl..

[19] Mir M., Ahmed N., Rehman A. (2017). Recent applications of PLGA based nanostructures in drug delivery. Colloids Surf. B.

[20] Gentile P., Chiono V., Carmagnola I., Hatton P.V. (2014). An overview of poly(lactic-co-glycolic) acid (PLGA)-based biomaterials for bone tissue engineering. Int. J. Mol. Sci..

[21] Ahmed O.A.A., El-Say K.M., Alahdal A.M. (2017). A PLGA-reinforced PEG in situ gel formulation for improved sustainability of hypoglycaemic activity of glimepiride in streptozotocin-induced diabetic rats. Sci. Rep..

[22] Shi Y., Sun X., Zhang L., Sun K., Li K., Li Y., Zhang Q. (2018). Fc-modified exenatide-loaded nanoparticles for oral delivery to improve hypoglycemic effects in mice. Sci. Rep..

[23] Kovarova M., Benhabbour S.R., Massud I., Spagnuolo R.A., Skinner B., Baker C.E., Sykes C., Mollan K.R., Kashuba A.D.M., García-Lerma J.G. (2018). Ultra-long-acting removable drug delivery system for HIV treatment and prevention. Nat. Commun..

[24] Zhang G., Luk B.T., Wei X., Campbell G.R., Fang R.H., Zhang L., Spector S.A. (2019). Selective cell death of latently HIV-infected CD4+ T cells mediated by autosis inducing nanopeptides. Cell Death Dis..

[25] Riitho V., Walters A.A., Somavarapu S., Lamp B., Rümenapf T., Krey T., Rey F.A., Oviedo-Orta E., Stewart G.R., Locker N. (2017). Design and evaluation of the immunogenicity and efficacy of a biomimetic particulate formulation of viral antigens. Sci. Rep..

[26] Le D.Q., Kuriakose A.E., Nguyen D.X., Nguyen K.T., Acharya S. (2017). Hybrid Nitric Oxide Donor and its Carrier for the Treatment of Peripheral Arterial Diseases. Sci. Rep..

[27] Liu H., Bao P., Li L., Wang Y., Xu C., Deng M., Zhang J., Zhao X. (2017). Pitavastatin nanoparticle-engineered endothelial progenitor cells repair injured vessels. Sci. Rep..

[28] Ma Y., Li J., Yao Y., Wei D., Wang R., Wu Q. (2016). A controlled double-duration inducible gene expression system for cartilage tissue engineering. Sci. Rep..

[29] Gwak S.J., Macks K., Bae S., Cecil N., Lee J.S. (2017). Physicochemical stability and transfection efficiency of cationic amphiphilic copolymer/pDNA polyplexes for spinal cord injury repair. Sci. Rep..

[30] Chen Y., Xu J., Huang Z., Yu M., Zhang Y., Chen H., Ma Z., Liao H., Hu J. (2017). An Innovative Approach for Enhancing Bone Defect Healing Using PLGA Scaffolds Seeded with Extracorporeal-shock-wave-treated Bone Marrow Mesenchymal Stem Cells (BMSCs). Sci. Rep..

[31] Díez-Martínez R., García-Fernández E., Manzano M., Martínez Á., Domenech M., Vallet-Regí M., García P. (2016). Auranofin-loaded nanoparticles as a new therapeutic tool to fight streptococcal infections. Sci. Rep..

[32] Fan Y., Zheng X., Ali Y., Berggren P.O., Loo S.C.J. (2019). Local release of rapamycin by microparticles delays islet rejection within the anterior chamber of the eye. Sci. Rep..

[33] Yang S., Traore Y., Jimenez C., Ho E.A. (2019). Autophagy induction and PDGFR-β knockdown by siRNA-encapsulated nanoparticles reduce *Chlamydia trachomatis* infection. Sci. Rep..

[34] Huang W., Tsui C.P., Tang C.Y., Gu L. (2018). Effects of Compositional Tailoring on Drug Delivery Behaviours of Silica Xerogel/Polymer Core-shell Composite Nanoparticles. Sci. Rep..

[35] Sousa F., Cruz A., Fonte P., Mendes Pinto I., Neves-Petersen M.T., Sarmento B. (2017). A new paradigm for antiangiogenic therapy through controlled release of bevacizumab from PLGA nanoparticles. Sci. Rep..

[36] Babu A., Amreddy N., Muralidharan R., Pathuri G., Gali H., Chen A., Zhao Y.D., Munshi A., Ramesh R. (2017). Chemodrug delivery using integrin-targeted PLGA-Chitosan nanoparticle for lung cancer therapy. Sci. Rep..

[37] Cui Y., Xu Q., Davoodi P., Wang D., Wang C. (2017). Enhanced intracellular delivery and controlled drug release of magnetic PLGA nanoparticles modified with transferrin. Acta Pharm. Sin..

[38] Yan S., Lu M., Ding X., Chen F., He X., Xu C., Zhou H., Wang Q., Hao L., Zou J. (2016). HematoPorphyrin Monomethyl Ether polymer contrast agent for ultrasound/photoacoustic dual-modality imaging-guided synergistic high intensity focused ultrasound (HIFU) therapy. Sci. Rep..

[39] Lee Y.H., Chang D.S. (2017). Fabrication, characterization, and biological evaluation of anti-HER2 indocyanine green-doxorubicin-encapsulated PEG-b-PLGA copolymeric nanoparticles for targeted photochemotherapy of breast cancer cells. Sci. Rep..

[40] Su X., Thomas R.G., Bharatula L.D., Kwan J.J. (2019). Remote targeted implantation of sound-sensitive biodegradable multi-cavity microparticles with focused ultrasound. Sci. Rep..

[41] Farzin A., Hassan S., Emadi R., Etesami S.A., Ai J. (2019). Comparative evaluation of magnetic hyperthermia performance and biocompatibility of magnetite and novel Fe-doped hardystonite nanoparticles for potential bone cancer therapy. Mater. Sci. Eng. C.

[42] Szalai A.J., Manivannan N., Kaptay G. (2019). Super-paramagnetic magnetite nanoparticles obtained by different synthesis and separation methods stabilized by biocompatible coatings. Colloids Surf. A Physicochem. Eng. Asp..

[43] Chaurasia A.K., Thorat N.D., Tandon A., Kim J.H., Park S.H., Kim K.K. (2016). Coupling of radiofrequency with magnetic nanoparticles treatment as an alternative physical antibacterial strategy against multiple drug resistant bacteria. Sci. Rep..

[44] Luo D., Poston R.N., Gould D.J., Sukhorukova G.B. (2019). Magnetically targetable microcapsules display subtle changes in permeability and drug release in response to a biologically compatible low frequency alternating magnetic field. Mater. Sci. Eng. C.

[45] Lengert E., Kozlova A., Pavlov A.M., Atkin V., Verkhovskii R., Kamyshinsky R., Demina P., Vasiliev A.L., Venig S.B., Bukreevaae T.V. (2019). Novel type of hollow hydrogel microspheres with magnetite and silver nanoparticles. Mater. Sci. Eng. C.

[46] Huang K.S., Yang C.H., Wang Y.C., Wang W.T., Lu Y.Y. (2019). Microfluidic Synthesis of Vinblastine-Loaded Multifunctional Particles for Magnetically Responsive Controlled Drug Release. Pharmaceutics.

[47] Popa E.G., Santo V.E., Rodrigues M.T., Gomes M.E. (2016). Magnetically-Responsive Hydrogels for Modulation of Chondrogenic Commitment of Human Adipose-Derived Stem Cells. Polymers.

[48] Miola M., Bellare A., Laviano F., Gerbaldo R., Vernéa E. (2019). Bioactive superparamagnetic nanoparticles for multifunctional composite bone cements. Ceram. Int..

[49] Minaei S.E., Khoei S., Khoee S., Vafashoar F., Mahabadi V.P. (2019). In vitro anti-cancer efficacy of multi-functionalized magnetite nanoparticles combining alternating magnetic hyperthermia in glioblastoma cancer cells. Mater. Sci. Eng. C.

[50] Rodrigues R.O., Baldi G., Doumett S., Garcia-Hevia L., Gallo J., Bañobre-López M., Dražić G., Calhelha R.C., Ferreira I.C.F.R., Lima R. (2018). Multifunctional graphene-based magnetic nanocarriers for combined hyperthermia and dual stimuli-responsive drug delivery. Mater. Sci. Eng. C.

[51] Peralta M.E., Jadhav S.A., Magnacca G., Scalarone D., Mártire D.O., Parolo M.E., Carlos L. (2019). Synthesis and in vitro testing of thermoresponsive polymer-grafted core-shell magnetic mesoporous silica nanoparticles for efficient controlled and targeted drug delivery. J. Colloid Interface Sci..

[52] Lim B.K., Tighe E.C., Kong S.D. (2019). The use of magnetic targeting for drug delivery into cardiac myocytes. J. Magn. Magn. Mater..

[53] Wei X., Liao J., Davoudi Z., Zheng H., Chen J., Li D., Xiong X., Yin Y., Yu X., Xiong J. (2018). Folate Receptor-Targeted and GSH-Responsive Carboxymethyl Chitosan Nanoparticles Containing Covalently Entrapped 6-Mercaptopurine for Enhanced Intracellular Drug Delivery in Leukemia. Mar. Drugs.

[54] Wang X., Yang L., Zhang H., Tian B., Li R., Hou X., Wei F. (2018). Fluorescent magnetic PEI-PLGA nanoparticles loaded with paclitaxel for concurrent cell imaging, enhanced apoptosis and autophagy in human brain cancer. Colloids Surf. B.

[55] Dalmina M., Pittella F., Sierra J.A., Souza G.R.R., Silva A.H., Pasa A.A., Creczynski-Pasa T.B. (2019). Magnetically responsive hybrid nanoparticles for in vitro siRNA delivery to breast cancer cells. Mater. Sci. Eng. C.

[56] Xu Y.H., Kim C.S., Saylor D.M., Koo D. (2017). Polymer degradation and drug delivery in PLGA-based drug-polymer applications: A review of experiments and theories. J. Biomed. Mater. Res. Part B Appl. Biomater..

[57] Chang D., Lim M., Goos J.A.C.M., Qiao R., Ng Y.Y., Mansfeld F.M., Jackson M., Davis T.P., Kavallaris M. (2018). Biologically Targeted Magnetic Hyperthermia: Potential and Limitations. Front. Pharmacol..

[58] Cazares-Cortes E., Cabana S., Boitard C., Nehlig E., Griffete N., Fresnais J., Wilhelm C., Abou-Hassan A., Ménager C. (2019). Recent insights in magnetic hyperthermia: From the “hot-spot” effect for local delivery to combined magneto-photo-thermia using magneto-plasmonic hybrids. Adv. Drug Deliv. Rev..

[59] Sahariah P., Másson M. (2017). Antimicrobial Chitosan and Chitosan Derivatives: A Review of the Structure–Activity Relationship. Biomacromolecules.

[60] Zeng T., Zhang Y., Yan Q., Huang Z., Zhang L., Yi X., Chen J., He G., Yin Y. (2017). Construction and in vitro evaluation of enzyme nanoreactors based on carboxymethyl chitosan for arginine deprivation in cancer therapy. Carbohydr. Polym..

[61] Islam S., Rahman Bhuiyan M.A., Islam M.N. (2017). Chitin and Chitosan: Structure, Properties and Applications in Biomedical Engineering. J. Polym. Environ..

[62] Wang Y., Beck-Broichsitter M., Yang M., Rantanen J., Bohr A. (2017). Investigation of nanocarriers and excipients for preparation of nanoembedded microparticles. Int. J. Pharm..

[63] Kenawy E.R., Abdel-Hay F.I., Tamer T.M., Abo-Elghit Ibrahim E.M., Mohy Eldin M.S. (2019). Antimicrobial activity of novel modified aminated chitosan with aromatic esters. Polym. Bull..

[64] Fioramonti Calixto G.M., de Annunzio S.R., Victorelli F.D., Frade M.L., Scanavez Ferreira P., Chorilli M., Fontana C.R. (2019). Chitosan-Based Drug Delivery Systems for Optimization of Photodynamic Therapy: A Review. AAPS Pharm. Sci. Tech..

[65] Kulikov S.N., Tikhonov V.E., Bezrodnykh E.A., Lopatin S.A., Varlamov V.P. (2015). Comparative evaluation of antimicrobial activity of oligochitosans against *Klebsiella pneumoniae*. Russ. J. Bioorganic Chem..

[66] Chien R.C., Yen M.T., Mau J.L. (2016). Antimicrobial and antitumor activities of chitosan from shiitake stipes, compared to commercial chitosan from crab shells. Carbohydr. Polym..

[67] Xia W., Liu P., Zhang J., Chen J. (2011). Biological activities of chitosan and chitooligosaccharides. Food Hydrocoll..

[68] Mansilla A.Y., Albertengo L., Rodriguez M.S., Debbaudt A., Zuniga A., Casalongue C.A. (2013). Evidence on antimicrobial properties and mode of action of a chitosan obtained from crustacean exoskeletons on *Pseudomonas syringae* pv. tomato DC3000. Appl. Microbiol. Biotechnol..

[69] Grumezescu A.M., Andronescu E., Holban A.M., Ficai A., Ficai D., Voicu G., Grumezescu V., Balaure P.C., Chifiriuc C.M. (2013). Water dispersible cross-linked magnetic chitosan beads for increasing the antimicrobial efficiency of aminoglycoside antibiotics. Int. J. Pharm..

[70] Holban A., Grumezescu V., Ficai A., Grumezescu A., Chifiriuc M., Iordache F., Andronescu E. (2016). Highly biocompatible magnetite nanoparticles functionalized with chitosan for improving the efficiency of antibiotics. Univ. Politeh. Buchar. Sci. Bull..

[71] Muñoz-Bonilla A., Echeverria C., Sonseca Á., Arrieta M.P., Fernández-García M. (2019). Bio-Based Polymers with Antimicrobial Properties towards Sustainable Development. Materials.

[72] Rainsford K.D. (2009). Ibuprofen: Pharmacology, efficacy and safety. Inflammopharmacology.

[73] Bushra R., Aslam N. (2010). An Overview of Clinical Pharmacology of Ibuprofen. Oman Med. J..

[74] Ahmetaj-Shala B., Tesfai A., Constantinou C., Leszczynski R., Chan M.V., Gashaw H., Galaris G., Mazi S., Warner T.D., Kirkby N.S. (2017). Pharmacological assessment of ibuprofen arginate on platelet aggregation and colon cancer cell killing. Biochem. Biophys. Res. Commun..

[75] Gkretsi V., Zacharia L.C., Stylianopoulos T. (2017). Targeting Inflammation to Improve Tumor Drug Delivery. Trends Cancer.

[76] Shah P.N., Marshall-Batty K.R., Smolen J.A., Tagaev J.A., Chen Q., Rodesney C.A., Le H.H., Gordon V.D., Greenberg D.E., Cannon C.L. (2018). Antimicrobial Activity of Ibuprofen against Cystic Fibrosis-Associated Gram-Negative Pathogens. Antimicrob. Agents Chemother..

[77] Chifiriuc M., Grumezescu A.M., Andronescu E., Ficai A., Cotar A.I., Grumezescu V., Bezirtzoglou E., Lazar V., Radulescu R. (2013). Water dispersible magnetite nanoparticles influence the efficacy of antibiotics against planktonic and biofilm embedded enterococcus faecalis cells. Anaerobe.

[78] Grumezescu A.M., Cristescu R., Chifiriuc M.C., Dorcioman G., Socol G., Mihailescu I.N., Mihaiescu D.E., Ficai A., Vasile O.R., Enculescu M. (2015). Fabrication of magnetite-based core–shell coated nanoparticles with antibacterial properties. Biofabrication.

[79] Grumezescu V., Holban A.M., Iordache F., Socol G., Mogoşanu G.D., Grumezescu A.M., Ficai A., Vasile B.Ş., Truşcă R., Chifiriuc M.C. (2014). MAPLE fabricated magnetite@eugenol and (3-hidroxybutyric acid-co-3-hidroxyvaleric acid)–polyvinyl alcohol microspheres coated surfaces with anti-microbial properties. Appl. Surf. Sci..

[80] Grumezescu V., Socol G., Grumezescu A.M., Holban A.M., Ficai A., Truşcǎ R., Bleotu C., Balaure P.C., Cristescu R., Chifiriuc M.C. (2014). Functionalized antibiofilm thin coatings based on PLA–PVA microspheres loaded with usnic acid natural compounds fabricated by MAPLE. Appl. Surf. Sci..

[81] Sunaric S., Petkovic M., Denic M., Mitic S., Pavlovic A. (2013). Determination of ibuprofen in combined dosage forms and cream by direct uv spectrophotometry after solid-phase extraction. Acta Poloniae Pharm. Drug Res..

[82] Yu-bin J., Xin L., Guo-song X., Qin-bing X. (2017). Detection and Analysis of the Quality of Ibuprofen Granules. IOP Conf. Ser. Earth Environ. Sci..

[83] Kokubo T., Kushitani H., Sakka S., Kitsugi T., Yamamuro T. (1990). Solutions able to reproduce in vivo surface-structure changes in bioactive glass-ceramic A-W. J. Biomed. Mater. Res..

[84] Yamaguchi S., Nath S., Matsushita T., Kokubo T. (2014). Controlled release of strontium ions from a bioactive Ti metal with a Ca-enriched surface layer. Acta Biomater..

[85] Andreu I., Natividad E. (2013). Accuracy of available methods for quantifying the heat power generation of nanoparticles for magnetic hyperthermia. Int. J. Hyperth..

[86] Saliev T., Feril L.B., Begimbetova D., Baiskhanova D., Klodzinskyi A., Bobrova X., Aipov R., Baltabayeva T., Tachibana K. (2017). Hyperthermia enhances bortezomib-induced apoptosis in human white blood cancer cells. J. Therm. Biol..

[87] Sun L., Cui Z.G., Zakki S.A., Feng Q.W., Li M.L., Inadera H. (2018). Mechanistic study of nonivamide enhancement of hyperthermia-induced apoptosis in U937 cells. Free Radic. Biol. Med..

[88] Brinker C.J., Frye G.C., Hurd A.J., Ashley C.S. (1991). Fundamentals of sol-gel dip coating. Thin Solid Films.

[89] Lu Y., Ganguli R., Drewien C.A., Anderson M.T., Brinker C.J., Gong W., Guo Y., Soyez H., Dunn B., Huang M.H. (1997). Continuous formation of supported cubic and hexagonal mesoporous films by sol-gel dip-coating. Nature.

[90] Kang B.S., Choi J.S., Lee S.E., Lee J.K., Kim T.H., Jang W.S., Tunsirikongkon A., Kim J.K., Park J.S. (2017). Enhancing the in vitro anticancer activity of albendazole incorporated into chitosan-coated PLGA nanoparticles. Carbohydr. Polym..

[91] Díaz E., Puerto I., Ribeiro S., Lanceros-Mendez S., Barandiarán J.M. (2017). The Influence of Copolymer Composition on PLGA/nHA Scaffolds’ Cytotoxicity and In Vitro Degradation. Nanomaterials.

[92] Qandil A.M., Obaidat A.A., Mohammed Ali M.A., Al-Taani B.M., Tashtoush B.M., Al-Jbour N.D., Al Remawi M.M., Al-Sou’od K.A., Badwan A.A. (2009). Investigation of the Interactions in Complexes of LowMolecular Weight Chitosan with Ibuprofen. J. Solution Chem..

[93] Acharya M., Mishra S., Sahoo R.N., Mallick S. (2017). Infrared Spectroscopy for Analysis of Co-processed Ibuprofen and Magnesium Trisilicate at Milling and Freeze Drying. Acta Chim. Slov..

[94] Vey E., Rodger C., Booth J., Claybourn M., Miller A.F., Saiani A. (2011). Degradation kinetics of poly(lactic-co-glycolic) acid block copolymer cast films in phosphate buffer solution as revealed by infrared and Raman spectroscopies. Polym. Degrad. Stabil..

[95] Sebri N.J.M., Amin K.A.M. (2016). Gellan Gum/Ibuprofen Hydrogel for Dressing Application: Mechanical Properties, Release Activity and Biocompatibility Studies. Int. J. Appl. Chem..

[96] Schwaminger S.P., Bauer D., Fraga-García P., Wagnerb F.E., Berensmeie S. (2017). Oxidation of magnetite nanoparticles: Impact on surface and crystal properties. CrystEngComm.

[97] Icriverzi M., Rusen L., Brajnicov S., Bonciu A., Dinescu M., Cimpean A., Evans W.R., Dinca V., Roseanu A. (2019). Macrophage in vitro Response on Hybrid Coatings Obtained by Matrix Assisted Pulsed Laser Evaporation. Coatings.

[98] AL-Janabi A.A.H.S. (2010). In Vitro Antibacterial Activity of Ibuprofen and Acetaminophen. J. Glob. Infect. Dis..

